# Medical Opinion and Movement

**Published:** 1908-03-28

**Authors:** 


### Medical Opinion and Movement.

WE have already referred to the investigation carried out by the medical officer of New York City on the relation between the propagation of flies from refuse and the spread of enteric fever and summer diarrhœa. An interesting report has been presented to the London County Council by one of their medical officers, Dr. W. H. Hamer, who has carried out some careful investigations to determine the effect of accumulations of refuse matter upon the number of flies in their vicinity. The results expressed in figures and graphic curves show clearly the importance of such accumulations as breeding-places for these insects. Of all refuse matter horse-dung is apparently the most directly implicated. Dr. Hamer draws attention to the close agreement between the curve representing the prevalence of flies and that representing the incidence of diarrhœa, but suggests that these "concomitant variations" may find an explanation in the dependence of both phenomena alike on the temperature of the soil. It seems, however, not unlikely that further investigations on these lines may show a direct causal connection between the prevalence of flies and the incidence of summer diarrhœa and possibly also of enteric fever.

ATOXYL appears to be rapidly winning the favour of French syphilologists as an antisyphilitic remedy. It is even claimed by some that its potency is equal to if not greater than that of the well-tried specifics mercury and potassium. Some of the cases recently published certainly support the view that we have in atoxyl a third highly useful weapon to fight the disease. It was the close similarity between the causal organisms of syphilis and sleeping sickness and the efficacy of atoxyl in the latter disease which suggested a trial of the drug in the treatment of syphilitic affections. Dr. Salmon reported favourably to the French Biological Society on its administration in several cases, and the subject was then taken up by others, notably by Dr. Hallopean, whose paper to the Academy of Medicine on the treatment of 120 cases has already received notice in these columns. A further communication has just appeared by M. Guiard, in which two remarkable cases of syphilis treated by this new remedy are recorded. In each of these cases secondary symptoms of a serious nature developed, and persisted in spite of mercurial treatment of a varied and most thorough nature. Commencing with administration by the mouth, this was followed by intramuscular injections of calomel and later of grey oil, without, however, any permanent arrest of the disease. It was only then that injections of atoxyl were resorted to; and the result was, according to M. Guiard, magical. After the first two or three injections the ulcerations and other symptoms showed definite signs of clearing, and in a short time all the lesions were completely healed, and the disease arrested. Such cases are highly interesting and convincing, inasmuch as mercury was first given a thorough trial and failed. Dr. Hallopean is very emphatic as to the dangers attending the use of the drug in too large doses. He advises injections of about 0.5 gramme of atoxyl at intervals of two to seven days.

THE medical profession in Denmark have just experienced the gratifying results of combination and unity. The hospitals in that country are under the control and management of the municipal authorities, and the medical staffs, both resident and visiting, are salaried officials. The remuneration has been, however, quite inadequate. In accordance with representations from the Association of Junior Medical Men the town council of Copenhagen were disposed to improve the conditions of service, but at the last moment these proposals were successfully opposed. The Association then, with the support of the Danish General Medical Association, invited all medical men to abstain from applying for appointments in the Copenhagen municipal hospitals, but offered to supply locum-tenentes at the usual rate of charge in order to insure the proper care of the sick. The whole profession showed themselves united on the question: no one applied for vacant appointments and considerable sympathy was evinced by the public and in the lay Press in their behalf. The Copenhagen Town Council have, therefore, seen fit to make a timely capitulation, and the conflict has ended with a victory for the Association. There is now yet another conflict in prospect. The State has assumed a sort of monopoly of the treatment of venereal diseases, and all persons are entitled to free treatment for these diseases. The law obliges municipalities to appoint and pay medical men for this purpose. The Association holds that remuneration should be made in the form of a fee for every consultation, and not by a fixed salary, and this is now the question at issue between the Association and the municipality of Copenhagen.

SPINAL anæsthesia is now favourably viewed by many surgeons for operations carried out on the lower part of the body, and there are not a few enthusiasts who claim for the method many advantages over the administration of a general anæsthetic. The safety of the method has been greatly increased by the introduction of such drugs as novococaine and stovaine. An interesting discussion took place at the last meeting of the Société de Chirurgie in Paris on this subject. M. Chaput gave the results of his experience at the Lariboisière Hospital. During last year 425 operations were performed with lumbar injections without a death. The chief danger is syncope, and this was avoided by the use of caffeine and by intravenous injections of serum. On the other hand M. Hartmann recorded two cases of sudden death from raising the pelvis, and presumably causing thereby a diffusion of the injected fluid towards the medulla. M. Guiard had experienced three fatal results, death taking place in each case some weeks after the operation from central softening. He had used the method in some hundreds of cases, but in consequence of these fatalities had now completely abandoned it. Mr. A. E. J. Barker, who is perhaps the greatest exponent of the method on this side, is of opinion that serious after-effects are entirely preventable by attention to details. The method certainly appears to require greater skill and experience than the administration of a general anæsthetic. It is hardly time to judge yet how far it can be considered a rival substitute for general anæsthesia, but, apart from comparative advantages and dangers, there is the personal factor of the patient to be considered. For those who would beg above all things that they should be rendered unconscious and "not know anything about it," general anæsthesia will always hold the field. On the other hand, there are patients who have an innate horror of a general anæsthetic, and for these lumbar injections offer a very suitable alternative.

David Lawson Walker, in a paper read before the Glasgow Eastern Medical Society, discusses the value of consumption sanatoria. More than five millions of deaths every year are due to pulmonary tuberculosis. Stevenson died of it; Keats, Schiller, Artemus Ward, Chopin, Laennec, Spinoza, Purcell, and Rachell were victims to it. As the author rightly says, the subject of consumption is one which, directly or indirectly, concerns every citizen, and which has for the medical profession, "whose high ideals embrace not merely the cure, but the eradication and prevention of disease," a very special and active interest. Recently the sanatorium treatment has engaged much attention, and it seems that the time has come for a critical discussion and examination of the claims and merits of that method of treatment. The question of the hour is, "Are consumptive sanatoria worth while?" Their record of work, in this country at least, warrants in the opinion of many the emphatic "No" which some sceptics have returned in answer to this query.

Dr. Walker puts the case for the detractors very forcibly and very fairly, but he points out where their objections fail. Thus there is the "historical objection"—that sanatoria are new and untried, and that they are "the latest fad of a faddy profession." But, as Dr. Walker shows, here, as in most other things, the saying *sub sole nil novi* holds good. Sanatorium treatment was advocated as early as 1747 (we fancy that Dr. Walker can find an even earlier date if he studies Paulus Eginetus), and the first consumption sanatorium was founded in 1840 by George Bodington, a Warwickshire general practitioner. To many it will be news to hear that England has been the pioneer in sanatorium construction, for the first continental sanatorium was only erected in 1859. Brehmer, the founder of the Goebersdorff establishment, which served a type for all the earlier German sanatoria, was far more enthusiastic than Bodington and exploited the sanatorium treatment for all it was worth. The other objections put forward by the sceptics are not less fully dealt with. The clinical objections, for example, are refuted by indubitable facts. There can be no doubt, for instance, that the sanatorium is a great educative force or that it exerts a "much more far-reaching influence in relationship to consumption than is effected by any fever hospital in relationship to the zymotic disease with which it deals." Its patients are proselytes, eager to spread the hygienic doctrines they have imbibed, and their cumulative influence cannot but be for good throughout the country. Nor can the efficiency of the sanatorium as a place of cure be any longer doubted. In Germany the percentage of "economic cures" has been steadily rising: in 1899 it was 74; now it stands at 82. These "economic cures" are a standing advertisement of the benefits of sanatorium treatment, and a standing refutation of the sceptics who say that such treatment is useless. The last objection—namely, that the treatment is extravagant—Dr. Walker discusses only briefly. That sanatoria are built on principles which are not strictly economical there can be as little doubt as that they are doing excellent work. Much money has been wasted on such establishments, and the charge of extravagance is well founded; but in time, when the novelty wears off and such institutions are built on business-like as well as up-to-date scientific lines, less will be heard of the prodigality of sanatorium advocates.

Finally Dr. Walker discusses the position which Great Britain, the country that took the lead in sanatorium work, at present holds in regard to this method of treatment. The supply of sanatoria in this country is very much below the demand, especially for poorer patients who cannot afford the comparatively high scale of fees that obtains at most of the private establishments. The public is daily becoming more alive to the benefits to be gained from sanatorium treatment in selected cases, and is thus emphatically replying in the affirmative to the question "Are sanatoria worth while?"

Dr. George Carpenter recently read a paper before the British Balneological and Climatological Society on the subject of "Seaside Treatment for Sick Children." He briefly reviewed various ailments for which seaside treatment is beneficial, chief among them being tuberculous diseases of the bones, joints, the lymph glands and peritoneum, and early phthisis. He said that as a tonic after surgical operations, and after recovery from the exanthemata, sea air is most valuable; and that cases well-nigh moribund from summer diarrhœa and entero-colitis derive the greatest benefit from it. He then passed in review the different seaside climates within a moderate train journey of London. He considered that the physicians practising at the seaside do not make the best use of the natural advantages of their towns—namely, the sea water. In this respect they are behind their Continental brethren. He advocated the study of the effects of hot and cold sea-water baths and douches on the secretions and metabolic processes of children, and of the therapeutic effects of such remedies on their complaints. He thought the medical men residing at the seashore resorts should give more information to physicians residing in other parts as to the peculiarities of the climates of their towns, and more information concerning the class of cases which would receive benefit.

In a recent number of The Hospital (August 10, 1907, p. 494) we drew attention to the encouraging results which had been obtained in the treatment of cases of surgical tuberculosis by means of injections of Baranek's tuberculin. Laasueur has recently reported further results in the treatment of lupus. Baranek's tuberculin is a mixture of bacillary toxins, and is obtainable in 10 cc. flasks. Minute quantities of this tuberculin were injected into the tissues surrounding the lupus patches, producing a local inflammatory reaction. The treatment extends over several weeks, the patients receiving two or three injections of the attenuated tuberculin every week. In the author's cases this treatment was followed by marked improvement and by complete cure in three cases. Baranek's tuberculin, as we have pointed out, is well worth the attention of English surgeons, and the simplicity of the treatment would seem to make it a valuable adjunct in the treatment of tuberculosis by the general practitioner.

Goldstein reports some unusual cases of retro-pharyngeal abscess. The first case on record, according to Morell Mackenzie, was reported by Galen, but in recent years the subject has been studied by many observers, and monographs have been written on it. The classical work of the Boakai's is known to every student of surgery, but the author complains that comparatively little attention has hitherto been paid to the ætiology of the affection. He reports three cases, which all offer material for interesting comment. In one, the patient being a six months old child, a diagnosis of lympho-sarcoma was made on the microscopical examination of a section of pharyngeal tissue, and a bad prognosis was given. Later on free opening and drainage of the abscess cavity effected a complete cure. The third case is doubly interesting, because there was not only a difficulty in diagnosis, but because the author decided finally to operate under local infiltration anæsthesia. The diagnosis in this case was rendered difficult owing to the impossibility of examining the little patient. Tracheotomy was performed and the retro-pharyngeal abscess afterwards opened, and the child made a good recovery.

In commenting on these three cases, Goldstein draws attention to the comparative rarity of retro-pharyngeal abscess in adult patients. In early childhood "there is an unusual activity of the lymphatic ring and of the lymphoid tissue in the pharynx, and there are several small, deeply located lymphatic glands imbedded in the connective tissue of the posterior pharynx. . . . It would be logical to assume, therefore, that when an acute infectious process takes place in the nose, naso-pharynx, accessory sinuses, ear, tongue, or larynx, a continuity of lymphoid tissue may readily carry such infection into the depths of the pharynx walls. If the presence of this lymphoid tract in the pharynx area is responsible for the frequency with which retro-pharyngeal abscess occurs in young children, then the scarcity of its occurrence as the age of the subject advances may also be conversely proven, for, with adolescence and maturity, the lymphoid tissue, which is so great a productive agency of hypertrophy or disease of any part of Waldeyer's ring, greatly atrophies, and the pathology depending on this tissue is of necessity lessened." The second case exemplifies the danger of making a diagnosis on purely microscopic examination of suspected tissues by a pathologist who has not seen the patient and is therefore ignorant of the clinical picture. The author, it may be mentioned, operates with the patient lying in the dorsal position, the head hanging down. He advocates deep and free incision into the pharyngeal wall, the bistoury being kept sharply in the mid-line to avoid damaging the internal carotid.

In a paper upon "The Therapeutics of Diet," read before the Therapeutical and Pharmacological Section of the Royal Society of Medicine, Dr. Harry Campbell reviewed the changes which man's diet has undergone since simian times. Man's simian ancestor was mainly frugivorous (*i.e.* he subsisted chiefly on a concentrated vegetable diet), but he also consumed a small proportion of animal food. Man in his evolution from the ape has made three great dietetic advances. The first advance was made when he learnt to hunt and fish, by which means he greatly increased his supply of animal food. The second advance consisted in the artificial preparation of his food. The third and final advance was cibi-culture—*i.e.* the breeding of animals and the cultivation of the vegetable kingdom for purposes of food. Dr. Campbell traced the fluctuations in the relative proportion of animal and vegetable food consumed by man during the various phases of his evolution, and contended that during the early hunting period he was even more carnivorous than vegetarian; and that inasmuch as man has evolved from the ape on a highly animalised diet, it is idle to contend that he is by nature essentially vegetarian and that animal food is necessarily injurious to him.

Dr. Campbell drew attention to the fact that since the advent of cookery man has consumed less and less raw vegetable food, and that during this time his cooked vegetable food had become increasingly soft, so much so, indeed, that from the dietetic standpoint the present age might be called "The Age of Pap." He insisted that a due proportion of vegetable food should be eaten raw, and that cooked farinaceous foods should be in a form compelling abundant mastication. Were this plan adopted, not only indigestion, but diseases of the teeth, nose and throat would greatly diminish in frequency. Dr. Campbell also drew attention to the enormous increase in the consumption of sugar within recent years. He pointed out that whereas wild honey was the sole source of pure sugar for primitive man, thousands of tons of cane sugar are at the present time extracted yearly from sugar cane and beetroot. He has no doubt that in England at all events far more good is to be obtained from curtailing starch and sugar than from cutting down the animal food, although the latter may sometimes be necessary also.

